# Characterization of Li–S Batteries Using Laboratory Sulfur X-ray Emission Spectroscopy

**DOI:** 10.1021/acsaem.0c02878

**Published:** 2021-02-23

**Authors:** Matjaž Kavčič, Marko Petric, Ava Rajh, Kristina Isaković, Alen Vizintin, Sara Drvarič Talian, Robert Dominko

**Affiliations:** † Jožef Stefan Institute, Jamova 39, 1000 Ljubljana, Slovenia; ‡ Faculty of Mathematics and Physics, University of Ljubljana, Jadranska 19, 1000 Ljubljana, Slovenia; § Faculty of Geotechnical Engineering, University of Zagreb, Varaždin 42000, Croatia; ∥ Jožef Stefan International Postgraduate School, Jamova 39, 1000 Ljubljana, Slovenia; ⊥ National Institute of Chemistry, Hajdrihova 19, 1000 Ljubljana, Slovenia; # Faculty of Chemistry and Chemical Technology, University of Ljubljana, Večna pot 113, 1000 Ljubljana, Slovenia

**Keywords:** X-ray emission spectroscopy, Li−S battery, tender X-ray range, sulfur cathode, MeV proton excitation, DFT

## Abstract

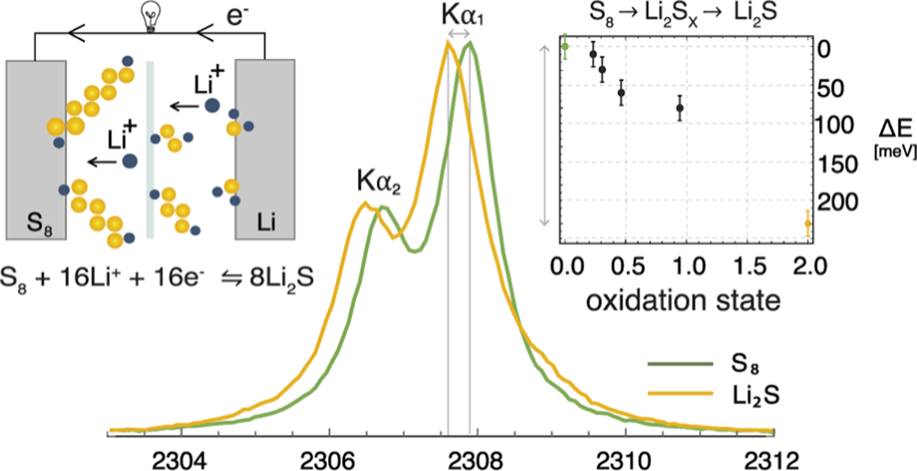

Application of laboratory-based X-ray analytical techniques that are capable of a reliable characterization of the chemical state of sulfur within bulk battery cathode in parallel with electrochemical characterization is essential for further development of lithium–sulfur batteries. In this work, MeV proton-induced X-ray emission (XES) sulfur measurements were performed in ex situ mode on laboratory-synthesized sulfur standards and precycled battery cathodes. The average sulfur charge was determined from the energy shift of the Kα emission line and from the spectral shape of the Kβ emission spectrum. Finally, operando Kα XES measurements were performed to monitor reduction of sulfur within battery cathode during discharge. The experimental approach presented here provides an important step toward more routine laboratory analysis of sulfur-based battery systems and also other sulfur-neighboring low-*Z* bulk materials with emission energies in the tender X-ray range.

## 1 Introduction

X-ray emission spectroscopy (XES) is emerging as a technique complementary to X-ray absorption spectroscopy (XAS), providing information on local electronic structure and bonding configuration of atoms within different bulk materials. Due to its specific properties, such as the capability of recording single-shot spectra required in time-resolved studies at free-electron lasers,^1^ ability to measure spin density,^2^ and identify different light ligands (C, N, O) in 3d transition-metal complexes,^3^ XES has been successfully applied in various research areas.^4,5^ Similar to XAS, XES studies are currently also restricted to large synchrotron facilities and more fundamental research. In particular, XES in the hard X-ray range has been extensively used to study 3d transition-metal complexes.^2,3,6−8^ The emission energy range of sulfur, however, remains out of reach for large in-air hard X-ray emission spectrometers used in these studies. Dedicated in-vacuum spectrometers are thus required in electronic structure studies of low-*Z* elements (including sulfur), which is the main reason why tender X-ray region has been much less explored. In our previous work, we have extended the range of XES studies also to tender X-ray range: we have used XES to study the electronic structure of sulfur in solid^9−11^ and liquid materials.^12^ The high scientific relevance of the tender X-ray range has been widely recognized, and several in-vacuum tender XES spectrometers have been developed recently.^13−17^

The main objective of this work is to explore the capabilities of sulfur XES as a laboratory analytical tool used to characterize electrochemical processes within lithium–sulfur (Li–S) batteries. These are currently one of the most promising options for the next-generation battery systems due to high energy density well beyond currently available Li-ion batteries.^18−20^ Operation of the Li–S battery is based on a simple reaction S_8_ + 16Li^+^ + 16e^–^ ↔ 8Li_2_S describing the reduction of sulfur at the cathode during discharge. However, the sulfur reduction mechanism is more complex and proceeds through the formation of long-chain lithium polysulfides, which are sequentially shortened before reaching the Li_2_S end discharge product. In contrast to the initial and final solid crystalline phases, intermediate polysulfides are highly soluble in the electrolyte. This introduces several problems that have so far prevented reaching a fully reversible theoretical capacity. At the moment, X-ray absorption spectroscopy (XAS) has been most commonly used to study the electrochemical conversion of sulfur within the working battery cell.^21−31^ However, all of these XAS studies are restricted to synchrotron facilities providing a monochromatic tunable photon beam. The obvious access limitations of synchrotron beamlines represent a bottleneck for routine sulfur XAS analysis across the electrochemical community. While, generally, X-ray absorption measurements can be performed in a laboratory, the available laboratory XAS setups^32−35^ are restricted to measurements in transmission mode in the hard X-ray energy range above 5 keV and are therefore not applicable for sulfur XAS studies of bulk materials. An XES setup consisting of a dedicated tender X-ray emission spectrometer coupled to a laboratory source of ionizing radiation has the potential to bring electronic and atomic structure analyses from the synchrotron facilities to smaller laboratories and enable analytical laboratory testing of Li–S batteries.

In this work, our tender X-ray emission spectrometer^36^ has been combined with MeV proton source to explore sulfur XES as a potential analytical tool used to characterize electrochemical processes within Li–S and other sulfur-based battery systems. The experimental approach employed in our previous electronic structure studies of several third-row elements in different compounds and materials^37−40^ has been used to examine the local electronic structure of sulfur within the Li–S battery cathode. First, we have recorded Kα and Kβ proton-induced XES spectra from different sulfur standards corresponding to sulfur compounds produced electrochemically within the battery. Measurements on the standards were used to analyze ex situ spectra recorded on precycled battery cathodes stopped at different points along the cycle. Finally, operando Kα XES spectra collected from a Li–S battery cathode were used to probe sulfur reduction during discharge.

## 2 Experimental Section

### 2.1 Preparation of the Li_2_S*_X_* Standards and a Cathode Composite

Polysulfides with different stoichiometric ratios between sulfur and lithium Li_2_S*_X_* (*X* = 2, 4, 6, 8) were synthesized by mixing the stoichiometric amounts of lithium metal scraps and sulfur in excess of dried tetrahydrofuran (THF). The mixture was continuously mixed in a closed flask inside an argon-filled glovebox at 50 °C until all of the materials were fully dissolved. To prepare solid targets suitable for XES measurements, the solvent was removed under reduced pressure, and the isolated powders were pressed into pellets (8 mm diameter) with boron nitride (BN) in a mass ratio of 80 wt % lithium polysulfides and 20 wt % BN. The pellets were sealed into a pouch bag with an 8 mm hole covered with a 6 μm thick mylar window. Besides the Li_2_S*_X_* samples, S_8_ and Li_2_S reference targets were prepared in the same way using standard material (both from Sigma-Aldrich; purum p.a., ≥99% and 99.98%, respectively).

The cathode composite was prepared from Ensaco 350G (Imerys) carbon ball milled for 30 min at 300 rpm with sulfur in a mass ratio of 1:2. The mixture was heated with a heating ramp of 0.2 °C min^–1^ to 155 °C, where it was kept for 5 h and cooled afterward down to room temperature at a rate of 0.5 °C min^–1^. The electrodes were prepared by mixing the carbon/sulfur composite (66 wt % of sulfur), poly(vinylidene fluoride) (PVdF) binder, and conductive multiwalled nanotubes (NTL, M-grade) in a mass ratio of 80:10:10. The slurry was prepared in *N*-methyl-2-pyrrolidine (NMP) and cast on carbon-coated Al foil. The electrodes were dried overnight at 50 °C. The typical sulfur loading on carbon-coated Al electrode was approximately 1.5 mg of S cm^–2^. A pouch-type two-electrode cell was prepared inside an argon-filled MBraun glovebox. The sulfur cathode (2 cm^2^ electrode) was separated from metallic lithium (FMC, 110 μm) anode with glass fiber separator (GF-A, Whatman, 260 μm) The electrolyte was 1 M lithium 2-trifluoromethyl-4,5-dicyanoimidazole (LiTDI) (Solvionic, 99.9%) in TEGDME:DOL (1:1 vol %) and was normalized to 15 μL per mass of sulfur. The batteries were cycled to different deep-of-discharge (DOD) potentials using a Bio-Logic VMP3 galvanostat/potentiostat at a current density of C/20 (83.6 mAg^–1^). The cells were opened and disassembled inside an argon-filled glovebox. The cathodes for the ex situ measurement were placed and sealed into a pouch cell with an 8 mm hole covered with a 6 μm thick mylar foil window.

### 2.2 Operando Battery Cell

For the operando XES measurements, the battery electrodes were prepared in 2-propanol by mixing the carbon/sulfur composite (66 wt % of sulfur), poly(tetrafluoroethylene) (PTFE) binder, and conductive multiwalled nanotubes (NTL, M-grade) in a mass ratio of 80:10:10. The self-standing electrodes (1.13 cm^2^) were pressed onto a carbon-coated Al mesh collector (3.14 cm^2^). The electrodes were dried for 1 h at 50 °C. The sulfur loading on self-standing electrodes was approximately 1.6 mg of S. The battery cell for the operando measurement was assembled in an argon-filled MBraun glovebox using the operando vacuum-tight Swagelok cell with a 6 μm thick mylar foil plated with 500 Å aluminum on the side facing the cathode.^41^ Briefly, the sulfur cathode was separated from metallic lithium (FMC) anode with a glass fiber separator (GF-D Whatman, 670 μm). The electrolyte was 1 M LiTDI (Solvionic, 99.9%) in TEGDME:DOL (1:1 vol %), and for the operando measurement, it was used in excess (80 μL per mass of sulfur). The battery was discharged to 1.5 V using a Bio-Logic SP-200 galvanostat/potentiostat at a current density of C/30 (55.7 mAg^–1^).

### 2.3 XES Measurements

High-energy-resolution XES measurements were performed at the 2 MV tandem ion accelerator of the Jožef Stefan Institute in Ljubljana. The X-ray emission was induced by a broad unfocused MeV proton beam, and the beam current on the sample was around 30–50 nA. Proton-induced X-ray emission spectra were recorded by the Johansson-type in-vacuum crystal spectrometer for XES spectroscopy in the tender X-ray range.^36^ The spectrometer operates in dispersive mode, which is achieved by positioning the target within the Rowland circle toward the analyzer crystal. In our experiment, the target was placed at a distance of 42 cm in front of the diffraction crystal. The first-order reflection of the Si(111) crystal was used, and the diffracted photons were detected by a thermoelectrically cooled (−40 °C) CCD camera of 770 × 1152 pixels with 22.5 × 22.5 μm^2^ pixel size. The position spectra recorded by the CCD were converted to energy scale relative to the Kα_1_ and Kβ_1,3_ emission lines of the S_8_ sample using the corresponding reference energies.^42^ The energy resolution was determined by fitting the model, composed of two Voigt functions, to the Kα_1,2_ spectrum measured on the S_8_ sample. The Lorentzian widths were fixed to the natural broadening of the Kα_1,2_ lines (Γ_K_ + Γ_L_2,3__ = 0.612 eV^43^). Best fit with experiment was obtained for the Gaussian width of 0.48 eV full width at half-maximum (FWHM) representing our final experimental resolution.

First, the Kα_1,2_ and Kβ_1,3_ XES spectra from reference standards (Li_2_S*_X_*, Li_2_S, and S_8_) were recorded, followed by the Kα_1,2_ and Kβ_1,3_ XES measurements on six battery cathodes stopped at different points during discharge. In these measurements, 2 MeV protons were used. The total acquisition time was between 500 and 900 s for the Kα_1,2_ and was increased to 1.5–2.5 h for the Kβ_1,3_ XES spectra. For the operando XES measurements, an in situ vacuum-tight Swagelok cell was mounted within the vacuum chamber of the spectrometer, and XES spectra emitted from the back of the battery cathode were recorded sequentially during one discharge. The main problem of the operando measurements is the damage induced by the incident ionizing radiation (MeV protons), predominantly the radiolysis of the electrolyte, which distorts the electrochemistry in the battery and prevents us to get a regular discharge curve. To this end, we have reduced the beam current in operando measurements by an order of magnitude, to 2–3 nA, and proton energy to 1 MeV. In this case, it was possible to get a regular discharge curve indicating proper electrochemistry, but due to significantly reduced count rate, it was only possible to record operando Kα XES spectra.

## 3 Results and Discussion

### 3.1 XES on Reference Standards

**Figure 1:**
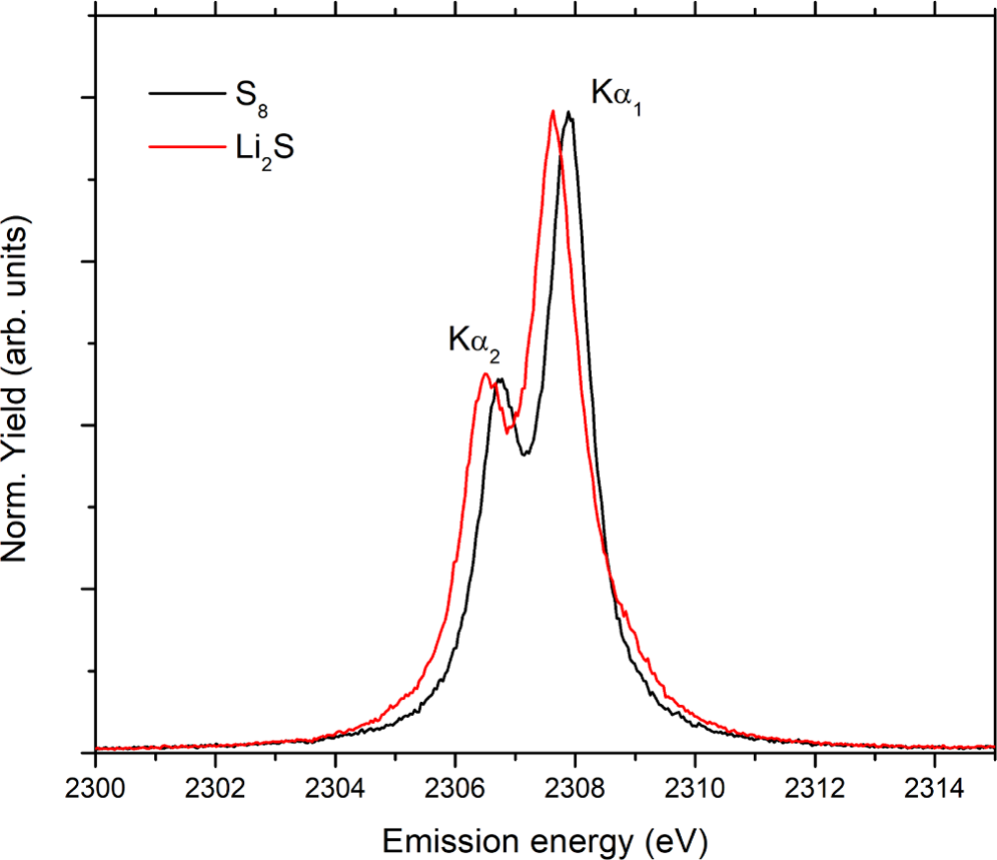
High-energy resolution proton-induced Kα_1,2_ sulfur X-ray emission spectra of Li_2_S and α-S_8_ standards.

**Figure 2:**
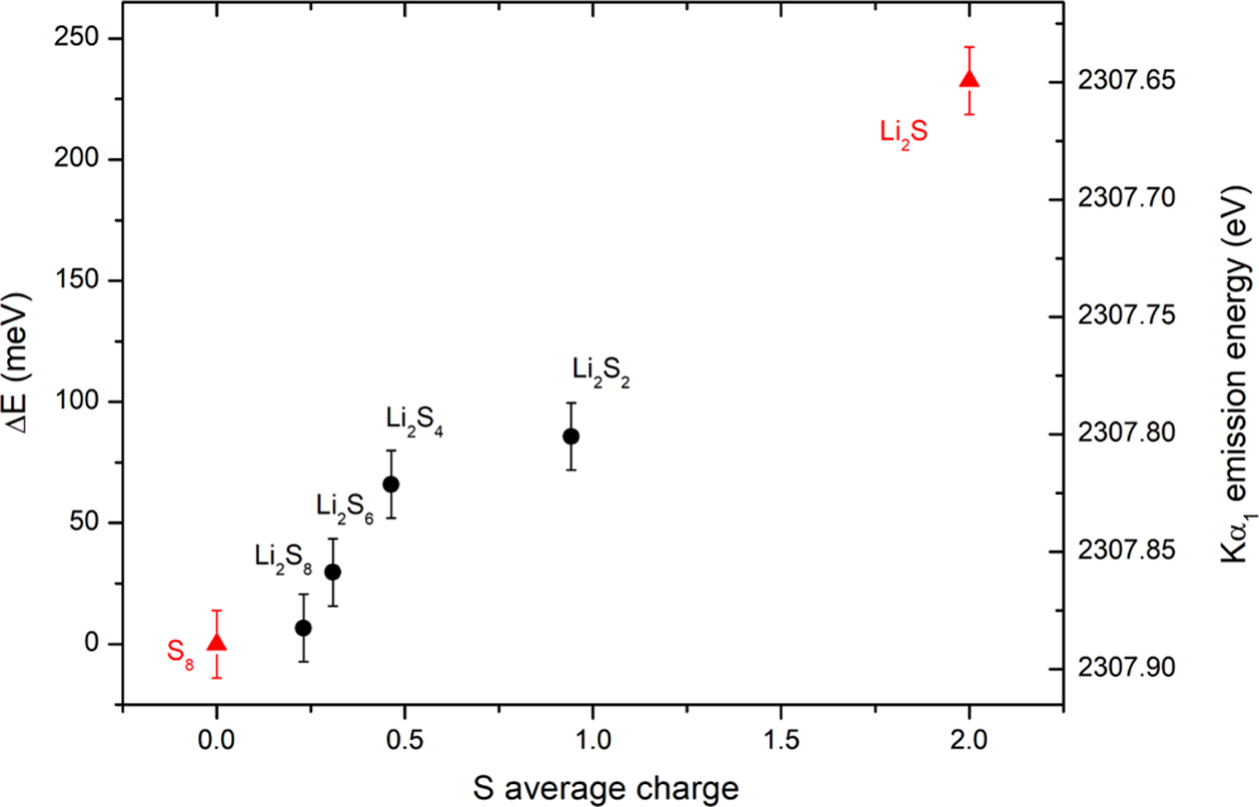
Experimental Kα_1_ absolute emission energies and energy shifts relative to the Kα_1_ energy of elemental sulfur as a function of the theoretical average sulfur charge.

The Kα emission line of sulfur corresponds to the 1s → 2p Core-to-Core (CtC) electron transition, which exhibits an atomic-like Lorentzian shape. Its characteristic doublet structure is due to the spin–orbit splitting of the 2p level. While the spectral shape of the Kα emission line is not influenced by the chemical environment of the sulfur atom, the spectrum exhibits tiny energy shifts, which are correlated with the local electronic charge.^9,38^Figure 1 shows the measured S Kα spectra of S_8_ and Li_2_S standards corresponding to the initial and final states of sulfur in the discharge process of the Li–S battery. To determine the Kα_1,2_ emission energies, the measured spectra were fitted with the model spectrum composed of two Voigt profiles (a convolution of a Lorentzian natural lineshape with the Gaussian function representing the spectrometer response function). In the case of the Li_2_S standard, an additional Voigt peak was introduced in the fitting model to account for the tiny shoulder observed on the high-energy tail of the Kα_1_ line. It was found that the sulfur 2p level spin–orbit splitting is independent of the chemical state, so we only use Kα_1_ emission line in further discussions. The energy shift between the measured spectra of the S_8_ and Li_2_S standards presented in Figure 1 corresponds to the difference between elemental and fully reduced (2^–^) sulfur. The emission energies of the measured Li_2_S*_X_* polysulfide standards are expected somewhere between the S_8_ and Li_2_S values. To obtain the average electronic charge of the sulfur atom within different Li_2_S*_X_* polysulfides, the ab initio quantum chemical calculations were performed using StoBe-deMon molecular/cluster density functional theory (DFT) code.^44^ The geometry optimization was first performed to obtain the ground state of each Li_2_S*_X_* molecule. In these calculations, we have used the TZVP (73111/6111/1) and DZVP (621/1/1) orbital basis sets^45^ for sulfur and lithium atoms, respectively. The generalized gradient approximation (GGA) exchange functional Be88^46^ and the GGA correlation functional PD91^47^ were used for the exchange–correlation part. The geometry optimization was followed by the Bader analysis,^48^ yielding the partial charge for each atom within the molecule. These partial charges were used to extract the average electronic charge on the S atom within the polysulfide molecule. The calculated values are tabulated in Table 1. Next, we have plotted the measured absolute emission energies and energy shifts relative to the S_8_Kα_1_ emission energy versus the theoretical average sulfur charge (Figure 2). A very nice correlation of the measured energy shift with the sulfur average charge is observed, in agreement with the general behavior of the CtC emission line of the third-row elements observed previously.^38^ While the measured shifts are below the natural core-hole broadening (sulfur Γ_K_ = 0.522 eV^43^), limiting ultimately the resolution of the measured spectrum, they are still significant enough to provide the average charge (oxidation state) of sulfur within the cathode of a Li–S battery.

**Table 1:** Calculated Average Charge of the S Atom within Different Li_2_S*_X_* Polysulfides

chemical formula	oxidation state	DFT theoretical S charge
α-S_8_	0	
Li_2_S_8_		–0.231
Li_2_S_6_		–0.309
Li_2_S_4_		–0.464
Li_2_S_2_		–0.942
Li_2_S	–2	

**Figure 3:**
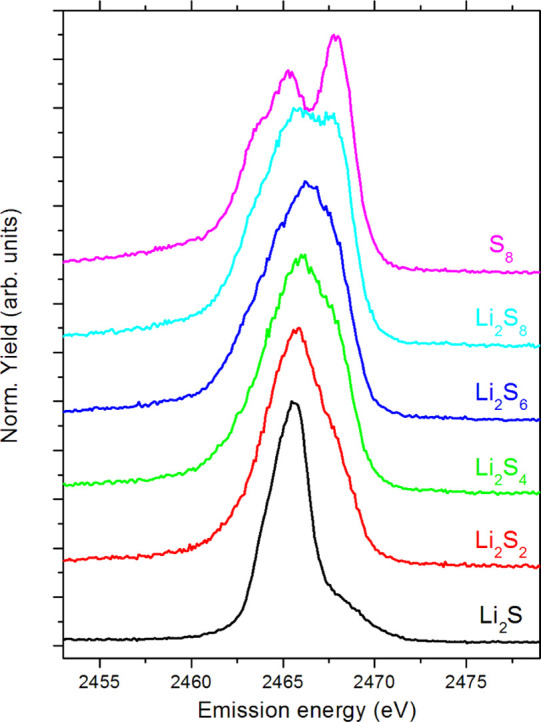
High-energy-resolution proton-induced Kβ_1,3_ sulfur X-ray emission spectra of sulfur standards measured in this work. The spectra are shifted vertically for clarity.

**Figure 4:**
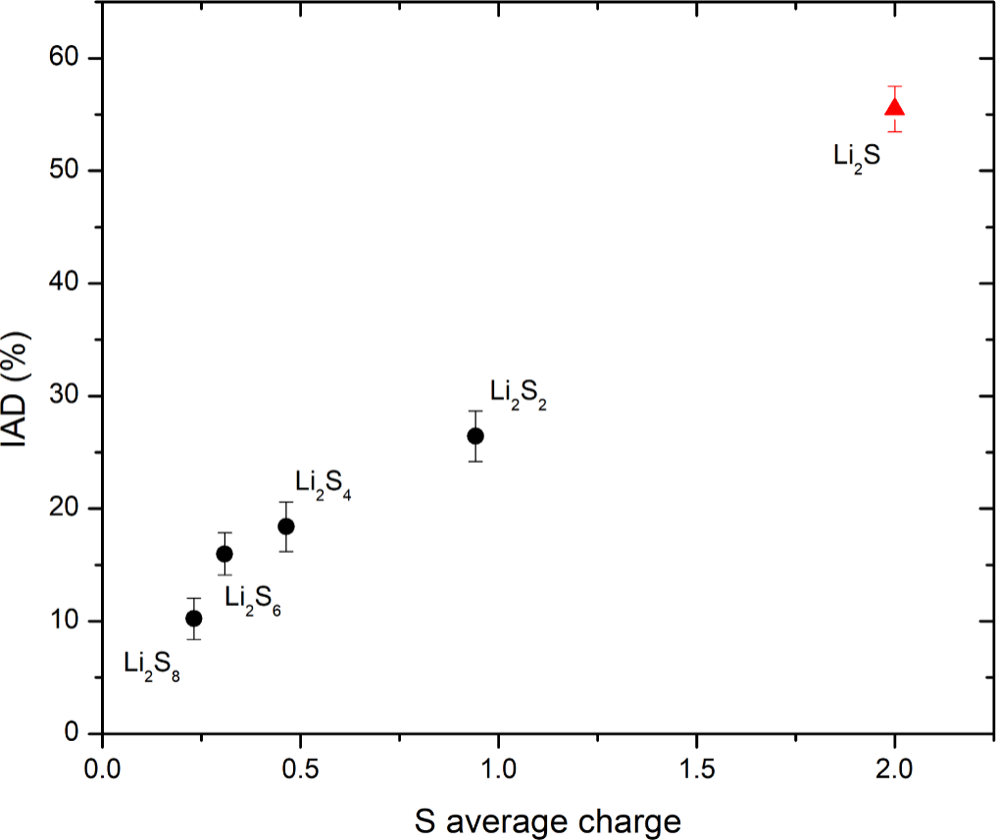
IAD of the measured Kβ emission spectra of Li_2_S_*X*_ standards relative to the reference spectrum of S_8_ plotted as a function of the sulfur average charge.

While the Kα energy shifts reflect the valence electronic population only indirectly by screening effects, higher sensitivity is expected in the Kβ valence-to-core (VtC) emission spectra corresponding to transitions from valence electronic states. The measured Kβ emission spectra of different standard compounds are presented in Figure 3, confirming high sensitivity to the sulfur valence electronic structure. The shape of the Kβ emission spectrum directly reflects the structure of the occupied valence molecular orbitals. A comprehensive theoretical modeling is required to interpret the measured Kβ emission spectrum and provide detailed information on the local electronic structure, bonding, and also local symmetry.^10^ However, in our analysis, we are looking for some rather simple spectroscopic parameter, which could be used to determine sulfur average charge within the Li–S battery cathode without the need for complex quantum chemistry calculations. One possibility used previously in the analysis of the Kβ emission spectra of the 3d transition metals is the integrated absolute difference (IAD).^49^ This is given as the absolute value of the difference between the sample spectrum and a reference spectrum, which are both normalized to integrated spectral area. In our case, the reference is the Kβ emission spectrum of pure elemental S_8_ corresponding to the initial state of sulfur within the Li–S battery cathode before discharge. The IAD is given by the following equation
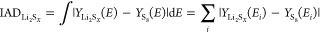
1The dependence of the IAD values for our measured Kβ emission spectra on the theoretical average sulfur charge is plotted in Figure 4. Also in this case, a very high correlation with sulfur average charge is found. Compared to the small energy shifts of the Kα emission lines, the Kβ emission spectra IAD values are relatively large, providing a high-sensitivity probe for the average sulfur charge. In addition, the IAD approach is model-independent, the analysis is straightforward, and can be performed on raw data without any complicated data treatment. The Kβ XES measurements therefore represent a very promising tool for the simple and robust determination of the average sulfur charge within the Li–S battery.

### 3.2 Ex Situ XES on Precycled Battery Cathodes

**Figure 5:**
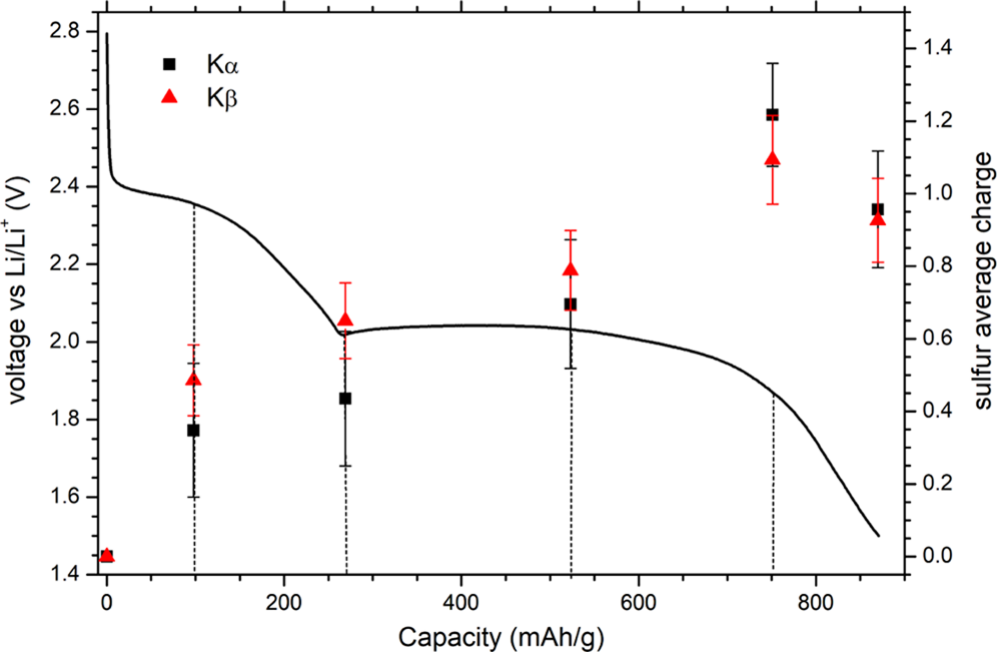
Sulfur average charge in individual battery cathodes stopped at different points during the discharge, as determined from the measured energy shift of the Kα_1_ emission line and from the changes in the shape of the Kβ emission spectra. The solid line represents the galvanostatic discharge curve with selected deep-of-discharge (DOD) points. A drop observed in the last point at the end of discharge indicates a possible polysulfide shuttle mechanism.

Both Kα and Kβ XES measurements on standards confirmed the high sensitivity to the sulfur average charge and can be used to determine the sulfur average oxidation state within the Li–S battery cathode. For that purpose, sulfur Kα and Kβ XES spectra were collected from separate battery cathodes, which were driven in parallel and stopped at different points along the electrochemical discharge curve (see Figure 5). First, the measured Kα spectra were used to determine the average sulfur charge from the energy shift of the measured Kα_1_ emission line relative to the emission energy of the Kα_1_ line recorded on the cathode in initial battery state, which corresponds to pure sulfur. In this process, the Kα_1_ energy shifts measured on standards were used (Figure 2). Generally, the laboratory-synthesized Li_2_S_*X*_ standards do not consist of pure stoichiometric mixtures, but are usually composed of multiple polysulfides with a mean Li_2_S_*X*_ length that differs from the nominal length (and therefore also average sulfur charge), especially for short polysulfides.^50^ The measured energy shifts for polysulfide standards are therefore used mainly to confirm high correlation of the Kα_1_ emission energy with the sulfur charge. For quantitative analysis, a linear interpolation of the S_8_ and Li_2_S measured values was used since these are the only two standards with a well-defined sulfur oxidation state (charge). This approach is supported by our previous Kα XES study of phosphorus-, sulfur-, and chlorine-containing compounds confirming a linear dependence of the Kα_1_ energy shift with the average charge of the central atom.^38^

In the next step, the collected Kβ spectra were used to calculate the corresponding IAD values using eq 1, with the Kβ spectrum from the cathode in the initial state used as a reference spectrum. The calculated IAD parameters were converted to the average sulfur charge. Also in this case, the IAD values on measured standards were used (Figure 4). For the reasons already discussed above for the analysis of Kα spectra, a linear interpolation of the S_8_ and Li_2_S IAD values was adopted, while the measurements of polysulfide standards were used merely to confirm the correlation between the IAD values and the sulfur average charge. The final sulfur average charges obtained from the Kα and Kβ emission spectra measured on several precycled battery cathodes are presented in Figure 5 together with the galvanostatic discharge curve. The two sets of average charge values are consistent with each other within the experimental uncertainty. The latter is slightly lower in the case of the Kβ emission spectra and is limited mainly by the statistical error of the collected spectra. The error could in principle be reduced further by increasing either the proton current or acquisition times, but this was avoided to reduce radiation damage of the cathode material. As expected, a gradual increase of the sulfur charge during battery discharge is observed, confirming the reduction of sulfur in the cathode. The absolute value at the end of the discharge does not reach the value of pure Li_2_S, indicating an incomplete sulfur redox conversion. This is consistent with the previous observation based on X-ray absorption measurements employing synchrotron radiation, where a significant amount of pure sulfur phase as well as some polysulfides were found in the battery cathode at the end of the discharge.^21,23^ It is also important to note that the measured XES spectra are not affected by target self-absorption. The latter represents an important obstacle in sulfur XAS measurements, which typically require diluted samples to avoid self-absorption-induced distortions of the measured spectra. The XES measurements presented here are performed on concentrated battery cathodes corresponding much closer to a realistic working battery system.

### 3.3 Operando XES

**Figure 6:**
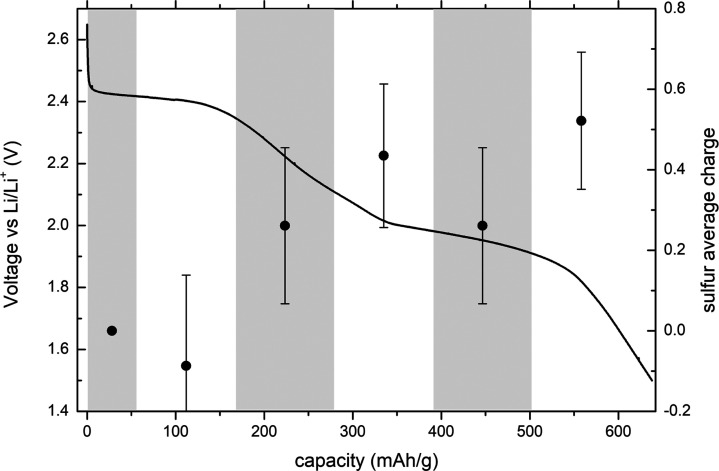
Sulfur average charge obtained from the operando Kα XES spectra measured during the battery discharge.

Ex situ measurements on precycled cathodes represent only the first approximation to the dynamics of the electrochemical sulfur conversion within the battery cathode during discharge. Even though they are run in parallel, the individual cathodes do not belong to exactly the same battery. In addition, the battery is dismantled to remove the cathode, which might cause disproportionation of polysulfides and some other small modifications of the chemical composition. The most genuine and accurate insight into the electrochemical conversion within battery cathode is therefore obtained in operando measurements on the working battery cell. The main problem of the operando measurements are the delicate electrochemical processes, which are perturbed by the irradiation with MeV energetic protons. Due to proton radiolysis of the electrolyte in the cell, it is very difficult to obtain a regular electrochemistry during proton irradiation. To limit this radiation-induced effect, we needed to reduce the proton energy to 1 MeV, as well as the incident proton current to a few nanoamperes (2–3 nA) dispersed over a large surface area. Because of the reduced energy and current of the incident protons, the count rate in XES spectra has dropped drastically and we were only able to collect Kα emission spectra over consecutive 2 h intervals during the battery discharge. The characteristic Kα_1_ peak emission energies were transformed to the sulfur charge averaged over the acquisition time using the conversion process described before for the analysis of ex situ XES measurements. The final results are presented in Figure 6 together with the corresponding electrochemical curve.

The high uncertainties in the final sulfur average charge values are a direct consequence of large statistical uncertainties in the measured Kα spectra resulting from extremely low count rates. Nevertheless, the gradual increase of the average sulfur charge during the battery discharge is clearly confirmed by our operando Kα XES measurements. The average charge at the end of the discharge is in this case significantly lower than in the case of ex situ measurements and points to a less efficient sulfur conversion. This is, in fact, suggested also by the electrochemical discharge curve with a less prominent and much narrower second plateau, as well as with a lower capacity reached at the end of the discharge compared to the one for ex situ measurements. Despite significant reduction of the incident proton dose, the electrochemistry is still slightly affected by the proton irradiation, resulting in a less representable case. Still, the example presented here can serve as a proof of principle, demonstrating the general capability of laboratory-based XES spectroscopy to address electrochemical processes within the Li–S battery cell.

## 4 Conclusions

Proton-induced sulfur XES spectroscopy was used to determine the sulfur average charge in the cathode of the Li–S battery. A series of sulfur Kα and Kβ emission spectra were recorded for individual compounds corresponding to intermediate products in the redox reaction within the battery cathode. Both the energy of the Kα emission lines and the spectral shape of the Kβ lines can be used to determine the oxidation state (average charge) of sulfur in the sample. Ex situ measurements on precycled cathodes provide a reliable insight into the consecutive reduction of sulfur during battery discharge. To perform sulfur chemical state analysis in parallel with electrochemical characterization, operando XES measurements were used. In this case, the accuracy of the analysis was limited by the very low count rate due to low incident proton dose, which is necessary to avoid perturbations of the electrochemical process by the incident beam. For that purpose, a more compact emission spectrometer with a small crystal bending radius could be used to increase the collection efficiency, and a different excitation source (X-ray tube) to reduce the radiation damage and further enhance the applicability of the method.

Since X-ray emission spectra are not sensitive to the excitation mode, the results presented here are directly applicable to XES combined with different laboratory sources used to produce initial core ionized atomic states. In this case, the access limitation inherent to synchrotron facilities is removed. XES thus opens the door to operando analysis on S-based battery systems and, in general, to laboratory-based characterization of bulk sulfur-containing materials.

## References

[ref1] Alonso-Mori R.; Kern J.; Gildea R. J.; Sokaras D.; Weng T.-C.; Lassalle-Kaiser B.; Tran R.; Hattne J.; Laksmono H.; Hellmich J.; Glöckner C.; Echols N.; Sierra R. G.; Schafer D. W.; Sellberg J.; Kenney C.; Herbst R.; Pines J.; Hart P.; Herrmann S.; Grosse-Kunstleve R. W.; Latimer M. J.; Fry A. R.; Messerschmidt M. M.; Miahnahri A.; Seibert M. M.; Zwart P. H.; White W. E.; Adams P. D.; Bogan M. J.; Boutet S.; Williams G. J.; Zouni A.; Messinger J.; Glatzel P.; Sauter N. K.; Yachandra V. K.; Yano J.; Bergmann U. Energy-dispersive X-ray emission spectroscopy using an X-ray free-electron laser in a shot-by-shot mode. Proc. Natl. Acad. Sci. U.S.A. 2012, 109, 19103–19107. 10.1073/pnas.1211384109. 23129631 PMC3511075

[ref2] Glatzel P.; Bergmann U. High resolution 1s core hole X-ray spectroscopy in 3d transition metal complexes—electronic and structural information. Coord. Chem. Rev. 2005, 249, 65–95. 10.1016/j.ccr.2004.04.011.

[ref3] Gallo E.; Glatzel P. Valence to Core X-ray Emission Spectroscopy. Adv. Mater. 2014, 26, 7730–7746. 10.1002/adma.201304994. 24861500

[ref4] Kern J.; Alonso-Mori R.; Tran R.; Hattne J.; Gildea R. J.; Echols N.; Glöckner C.; Hellmich J.; Laksmono H.; Sierra R. G.; Lassalle-Kaiser B.; Koroidov S.; Lampe A.; Han G.; Gul S.; DiFiore D.; Milathianaki D.; Fry A. R.; Miahnahri A.; Schafer D. W.; Messerschmidt M.; Seibert M. M.; Koglin J. E.; Sokaras D.; Weng T.-C.; Sellberg J.; Latimer M. J.; Grosse-Kunstleve R. W.; Zwart P. H.; White W. E.; Glatzel P.; Adams P. D.; Bogan M. J.; Williams G. J.; Boutet S.; Messinger J.; Zouni A.; Sauter N. K.; Yachandra V. K.; Bergmann U.; Yano J. Simultaneous Femtosecond X-ray Spectroscopy and Diffraction of Photosystem II at Room Temperature. Science 2013, 340, 491–495. 10.1126/science.1234273. 23413188 PMC3732582

[ref5] Lin J.-F.; Vankó G.; Jacobsen S. D.; Iota V.; Struzhkin V. V.; Prakapenka V. B.; Kuznetsov A.; Yoo C.-S. Spin Transition Zone in Earth’s Lower Mantle. Science 2007, 317, 1740–1743. 10.1126/science.1144997. 17885134

[ref6] Smolentsev G.; Soldatov A. V.; Messinger J.; Merz K.; Weyhermüller T.; Bergmann U.; Pushkar Y.; Yano J.; Yachandra V. K.; Glatzel P. X-ray Emission Spectroscopy To Study Ligand Valence Orbitals in Mn Coordination Complexes. J. Am. Chem. Soc. 2009, 131, 13161–13167. 10.1021/ja808526m. 19663435 PMC2752666

[ref7] Lancaster K. M.; Roemelt M.; Ettenhuber P.; Hu Y.; Ribbe M. W.; Neese F.; Bergmann U.; DeBeer S. X-ray Emission Spectroscopy Evidences a Central Carbon in the Nitrogenase Iron-Molybdenum Cofactor. Science 2011, 334, 974–977. 10.1126/science.1206445. 22096198 PMC3800678

[ref8] Lee N.; Petrenko T.; Bergmann U.; Neese F.; DeBeer S. Probing Valence Orbital Composition with Iron Kβ X-ray Emission Spectroscopy. J. Am. Chem. Soc. 2010, 132, 9715–9727. 10.1021/ja101281e. 20578760

[ref9] Alonso Mori R.; Paris E.; Giuli G.; Eeckhout S. G.; Kavčič M.; Žitnik M.; Bučar K.; Pettersson L. G. M.; Glatzel P. Electronic Structure of Sulfur Studied by X-ray Absorption and Emission Spectroscopy. Anal. Chem. 2009, 81, 6516–6525. 10.1021/ac900970z.

[ref10] Mori R. A.; Paris E.; Giuli G.; Eeckhout S. G.; Kavčič M.; Žitnik M.; Bučar K.; Pettersson L. G. M.; Glatzel P. Sulfur-Metal Orbital Hybridization in Sulfur-Bearing Compounds Studied by X-ray Emission Spectroscopy. Inorg. Chem. 2010, 49, 6468–6473. 10.1021/ic100304z. 20553025

[ref11] McGann O.; Bingham P.; Hand R.; Gandy A.; Kavčič M.; Žitnik M.; Bučar K.; Edge R.; Hyatt N. The effects of γ-radiation on model vitreous wasteforms intended for the disposal of intermediate and high level radioactive wastes in the United Kingdom. J. Nucl. Mater. 2012, 429, 353–367. 10.1016/j.jnucmat.2012.04.007.

[ref12] Niskanen J.; Sahle C. J.; Ruotsalainen K. O.; Müller H.; Kavčič M.; Žitnik M.; Bučar K.; Petric M.; Hakala M.; Huotari S. Sulphur Kβ emission spectra reveal protonation states of aqueous sulfuric acid. Sci. Rep. 2016, 6, 21012 10.1038/srep21012. 26888159 PMC4757876

[ref13] Holden W. M.; Hoidn O. R.; Ditter A. S.; Seidler G. T.; Kas J.; Stein J. L.; Cossairt B. M.; Kozimor S. A.; Guo J.; Ye Y.; Marcus M. A.; Fakra S. A compact dispersive refocusing Rowland circle X-ray emission spectrometer for laboratory, synchrotron, and XFEL applications. Rev. Sci. Instrum. 2017, 88, 073904 10.1063/1.4994739. 28764488

[ref14] Abraham B.; Nowak S.; Weninger C.; Armenta R.; Defever J.; Day D.; Carini G.; Nakahara K.; Gallo A.; Nelson S.; Nordlund D.; Kroll T.; Hunter M. S.; van Driel T.; Zhu D.; Weng T.-C.; Alonso-Mori R.; Sokaras D. A high-throughput energy-dispersive tender X-ray spectrometer for shot-to-shot sulfur measurements. J. Synchrotron Radiat. 2019, 26, 629–634. 10.1107/S1600577519002431. 31074425 PMC6510194

[ref15] Kuzmenko D.; Vogelsang U.; Hitz S.; Müller D.; Clark A. H.; Kinschel D.; Czapla-Masztafiak J.; Milne C.; Szlachetko J.; Nachtegaal M. A von Hamos spectrometer for in situ sulfur speciation by non-resonant sulfur Kα emission spectroscopy. J. Anal. At. Spectrom. 2019, 34, 2105–2111. 10.1039/C9JA00195F.

[ref16] Nowak S. H.; Armenta R.; Schwartz C. P.; Gallo A.; Abraham B.; Garcia-Esparza A. T.; Biasin E.; Prado A.; Maciel A.; Zhang D.; Day D.; Christensen S.; Kroll T.; Alonso-Mori R.; Nordlund D.; Weng T.-C.; Sokaras D. A versatile Johansson-type tender X-ray emission spectrometer. Rev. Sci. Instrum. 2020, 91, 033101 10.1063/1.5121853. 32259983

[ref17] Rovezzi M.; Harris A.; Detlefs B.; Bohdan T.; Svyazhin A.; Santambrogio A.; Degler D.; Baran R.; Reynier B.; Noguera Crespo P.; Heyman C.; Van der Kleij H.-P.; Van Vaerenbergh P.; Marion P.; Vitoux H.; Lapras C.; Verbeni R.; Kocsis M. M.; Manceau A.; Glatzel P. TEXS: in-vacuum tender X-ray emission spectrometer with 11 Johansson crystal analyzers. J. Synchrotron Radiat. 2020, 27, 813–826. 10.1107/S160057752000243X. 32381786 PMC7285681

[ref18] Evers S.; Nazar L. F. New Approaches for High Energy Density Lithium-Sulfur Battery Cathodes. Acc. Chem. Res. 2013, 46, 1135–1143. 10.1021/ar3001348. 23054430

[ref19] Manthiram A.; Fu Y.; Su Y.-S. Challenges and Prospects of Lithium–Sulfur Batteries. Acc. Chem. Res. 2013, 46, 1125–1134. 10.1021/ar300179v. 23095063

[ref20] Ji X.; Lee K. T.; Nazar L. F. A highly ordered nanostructured carbon sulphur cathode for lithium sulphur batteries. Nat. Mater. 2009, 8, 500–506. 10.1038/nmat2460. 19448613

[ref21] Cuisinier M.; Cabelguen P.-E.; Evers S.; He G.; Kolbeck M.; Garsuch A.; Bolin T.; Balasubramanian M.; Nazar L. F. Sulfur Speciation in Li-S Batteries Determined by Operando X-ray Absorption Spectroscopy. J. Phys. Chem. Lett. 2013, 4, 3227–3232. 10.1021/jz401763d.

[ref22] Patel M. U. M.; Arčon I.; Aquilanti G.; Stieviano L.; Mali G.; Dominko R. X-ray Absorption Near-Edge Structure and Nuclear Magnetic Resonance Study of the Lithium-Sulfur Battery and its Components. ChemPhysChem 2014, 15, 894–904. 10.1002/cphc.201300972. 24497200

[ref23] Dominko R.; Patel M. U. M.; Lapornik V.; Vizintin A.; Koželj M. N.; Tušar N.; Arčon I.; Stievano L.; Aquilanti G. Analytical Detection of Polysulfides in the Presence of Adsorption Additives by Operando X-ray Absorption Spectroscopy. J. Phys. Chem. C 2015, 119, 19001–19010. 10.1021/acs.jpcc.5b05609.

[ref24] Gao J.; Lowe M. A.; Kiya Y.; Abru̅na H. D. Effects of Liquid Electrolytes on the Charge-Discharge Performance of Rechargeable Lithium/Sulfur Batteries: Electrochemical and in-Situ X-ray Absorption Spectroscopic Studies. J. Phys. Chem. C 2011, 115, 25132–25137. 10.1021/jp207714c.

[ref25] Lowe M. A.; Gao J.; Abruna H. D. Mechanistic Insights into Operational Lithium-Sulfur Batteries by in Situ X-ray Diffraction and Absorption Spectroscopy. RSC Adv. 2014, 4, 18347–18353. 10.1039/c4ra01388c.

[ref26] Pang Q.; Kundu D.; Cuisinier M.; Nazar L. F. Surface-Enhanced Redox Chemistry of Polysulphides on a Metallic and Polar Host for Lithium-Sulphur Batteries. Nat. Commun. 2014, 5, 4759 10.1038/ncomms5759. 25154399

[ref27] Cuisinier M.; Cabelguen P.-E.; Adams B. D.; Garsuch A.; Balasubramanian M.; Nazar L. F. Unique Behaviour of Nonsolvents for Polysulphides in Lithium-Sulphur Batteries. Energy Environ. Sci. 2014, 7, 2697–2705. 10.1039/C4EE00372A.

[ref28] Gorlin Y.; Siebel A.; Piana M.; Huthwelker T.; Jha H.; Monsch G.; Kraus F.; Gasteiger H. A.; Tromp M. Operando Characterization of Intermediates Produced in a Lithium-Sulfur Battery. J. Electrochem. Soc. 2015, 162, A1146–A1155. 10.1149/2.0081507jes.

[ref29] Gorlin Y.; Patel M. U. M.; Freiberg A.; He Q.; Piana M.; Tromp M.; Gasteiger H. A. Understanding the Charging Mechanism of Lithium-Sulfur Batteries Using Spatially Resolved Operando X-Ray Absorption Spectroscopy Batteries and Energy Storage. J. Electrochem. Soc. 2016, 163, A930–A939. 10.1149/2.0631606jes.

[ref30] Vizintin A.; Chabanne L.; Tchernychova E.; Arčon I.; Stievano L.; Aquilanti G.; Antonietti M.; Fellinger T.-P.; Dominko R. The mechanism of Li2S activation in lithium-sulfur batteries: Can we avoid the polysulfide formation. J. Power Sources 2017, 344, 208–217. 10.1016/j.jpowsour.2017.01.112.

[ref31] Zhang L.; Sun D.; Feng J.; Cairns E. J.; Guo J. Revealing the Electrochemical Charging Mechanism of Nanosized Li2S by in Situ and Operando X-ray Absorption Spectroscopy. Nano Lett. 2017, 17, 5084–5091. 10.1021/acs.nanolett.7b02381. 28731713

[ref32] Taguchi T.; Harada J.; Kiku A.; Tohji K.; Shinoda K. Development of a new in-laboratory XAFS apparatus based on new concept. J. Synchrotron Radiat. 2001, 8, 363–365. 10.1107/S0909049500018458. 11512781

[ref33] Seidler G. T.; Mortensen D. R.; Remesnik A. J.; Pacold J. I.; Ball N. A.; Barry N.; Styczinski M.; Hoidn O. R. A laboratory-based hard X-ray monochromator for high-resolution X-ray emission spectroscopy and X-ray absorption near edge structure measurements. Rev. Sci. Instrum. 2014, 85, 113906 10.1063/1.4901599. 25430123

[ref34] Schlesiger C.; Anklamm L.; Stiel H.; Malzer W.; Kanngieser B. XAFS spectroscopy by an X-ray tube based spectrometer using a novel type of HOPG mosaic crystal and optimized image processing. J. Anal. At. Spectrom. 2015, 30, 1080–1085. 10.1039/C4JA00303A.

[ref35] Németh Z.; Szlachetko J.; Bajnóczi G.; Vankó G. Laboratory von Hámos X-ray spectroscopy for routine sample characterization. Rev. Sci. Instrum. 2016, 87, 103105 10.1063/1.4964098. 27802722

[ref36] Kavčič M.; Budnar M.; Mühleisen A.; Gasser F.; Žitnik M.; Bučar K.; Bohinc R. Design and performance of a versatile curved-crystal spectrometer for high-resolution spectroscopy in the tender X-ray range. Rev. Sci. Instrum. 2012, 83, 033113 10.1063/1.3697862. 22462912

[ref37] Petric M.; Bohinc R.; Bučar K.; Žitnik M.; Szlachetko J.; Kavčič M. Chemical State Analysis of Phosphorus Performed by X-ray Emission Spectroscopy. Anal. Chem. 2015, 87, 5632–5639. 10.1021/acs.analchem.5b00782. 25927339

[ref38] Petric M.; Kavčič M. Chemical speciation via X-ray emission spectroscopy in the tender X-ray range. J. Anal. At. Spectrom. 2016, 31, 450–457. 10.1039/C5JA00394F.

[ref39] Petric M.; Bohinc R.; Bučar K.; Nowak S. H.; Žitnik M.; Kavčič M. Electronic Structure of Third-Row Elements in Different Local Symmetries Studied by Valence-to-Core X-ray Emission Spectroscopy. Inorg. Chem. 2016, 55, 5328–5336. 10.1021/acs.inorgchem.6b00237. 27176734

[ref40] Kavčič M.; Petric M.; Vogel-Mikuš K. Chemical speciation using high energy resolution PIXE spectroscopy in the tender X-ray range. Nucl. Instrum. Methods Phys. Res., Sect. B 2018, 417, 65–69. 10.1016/j.nimb.2017.06.009.

[ref41] Kavčič M.; Bučar K.; Petric M.; Žitnik M.; Arčon I.; Dominko R.; Vizintin A. Operando Resonant Inelastic X-ray Scattering: An Appropriate Tool to Characterize Sulfur in Li-S Batteries. J. Phys. Chem. C 2016, 120, 24568–24576. 10.1021/acs.jpcc.6b06705.

[ref42] Deslattes R. D.; Kessler E. G.; Indelicato P.; de Billy L.; Lindroth E.; Anton J. X-ray transition energies: new approach to a comprehensive evaluation. Rev. Mod. Phys. 2003, 75, 35–99. 10.1103/RevModPhys.75.35.

[ref43] Campbell J.; Papp T. WIDTHS OF THE ATOMIC K–N7 LEVELS. At. Data Nucl. Data Tables 2001, 77, 1–56. 10.1006/adnd.2000.0848.

[ref44] Hermann K.; Pettersson L.; Casida M.; Daul C.; Goursot A.; Koester A.; Proynov E.; St-Amant A.; Salahub D.; Carravetta V.; Duarte H.; Friedrich C.; Godbout N.; Guan J.; Jamorski C.; Leboeuf M.; Leetmaa M.; Nyberg M.; Patchkovskii S.; Pedocchi L.; Sim F.; Triguero L.; Vela A. StoBe-deMon version 3.1. http://www.fhi-berlin.mpg.de/KHsoftware/StoBe/index.html 2011.

[ref45] Godbout N.; Salahub D. R.; Andzelm J.; Wimmer E. Optimization of Gaussian-type basis sets for local spin density functional calculations. Part I. Boron through neon, optimization technique and validation. Can. J. Chem. 1992, 70, 560–571. 10.1139/v92-079.

[ref46] Becke A. Density-functional exchange-energy approximation with correct asymptotic behavior. Phys. Rev. A 1988, 38, 3098–3100. 10.1103/PhysRevA.38.3098. 9900728

[ref47] Perdew J.; Chevary J.; Vosko S.; Jackson K.; Pederson M.; Singh D.; Fiolhais C. Atoms, molecules, solids, and surfaces: Applications of the generalized gradient approximation for exchange and correlation. Phys. Rev. B 1992, 46, 6671–6687. 10.1103/PhysRevB.46.6671. 10002368

[ref48] Bader R. F. W. A quantum theory of molecular structure and its applications. Chem. Rev. 1991, 91, 893–928. 10.1021/cr00005a013.

[ref49] Vankó G.; Rueff J.-P.; Mattila A.; Németh Z.; Shukla A. Temperature- and pressure-induced spin-state transitions in LaCoO_3_. Phys. Rev. B 2006, 73, 024424 10.1103/PhysRevB.73.024424.

[ref50] Robba A.; Barchasz C.; Bučar K.; Petric M.; Žitnik M.; Kvashnina K.; Vaughan G. B. M.; Bouchet R.; Alloin F.; Kavčič M. Fingerprinting Mean Composition of Lithium Polysulfide Standard Solutions by Applying High-Energy Resolution Fluorescence Detected X-ray Absorption Spectroscopy. J. Phys. Chem. Lett. 2020, 11, 5446–5450. 10.1021/acs.jpclett.0c01120. 32584577

